# Occupational, environmental and individual factors affecting the selection of winter clothing by open-pit miners in Barents region - a questionnaire and field study

**DOI:** 10.1186/2046-7648-4-S1-A135

**Published:** 2015-09-14

**Authors:** Kirsi Jussila, Sirkka Rissanen, Satu Mänttäri, Juha Oksa, Hannu Rintamäki, MineHealth project members

**Affiliations:** 1 Finnish Institute of Occupational Health, Oulu, Finland

## Introduction

Working in open-pit mines in the Barents region requires protection against cold, occasionally extreme temperatures and high wind speed. Employers are required to provide outer cold protective clothing for workers in outdoor tasks. Some companies also offer inner or middle clothing layers for voluntary use. However, workers use their own selection of winter garments underneath the outer clothing. This study aimed to evaluate the selection criteria of winter clothing of open-pit miners and its thermal protection at work.

## Methods

A questionnaire study (n = 1104) evaluating open-pit miners' cold experiences and the use of clothing in different ambient conditions was performed in three different open-pit mines in Northern Finland, Sweden and Russia among workers with duties consisting mainly of outdoor work. Basic thermal insulation (I_cl_) of the reported clothing ensembles was estimated by using the standard ISO 9920 (2007). Moreover, a field study was carried out in the two open-pit mines in Northern Finland and Sweden to be able to determine users' experiences and thermal insulation of the clothing (n = 14) by measuring ambient (T_a_) and skin temperatures (T_sk_), and dry heat loss from the skin during a typical work shift.

## Results

The questionnaire study showed that the I_cl _of the winter clothing was on an average 1.2 clo (0.186 m²K.W^-1^) and 1.5 clo (0.233 m²K.W^-1^) in mild wet cold (T_a _-5 to +5 °C) and dry cold (-20 to -10 °C) conditions, respectively. The clothing was selected based on cold exposure time, work load, environmental conditions and individual sensitivity to cold (p < 0.05). If wind and external moisture were experienced as a problem, higher clothing insulation was reported. Similarly, if thermal sensation was perceived cold on the whole body, fingers or toes, higher I_cl _of the clothing was selected in the both temperature ranges. Whereas, lower I_cl _of the clothing was reported, if workers were sweating often at work. In addition, the I_cl _was higher than average on persons who reported cold related symptoms or pains in the cold weather. In the field experiment the measured mean I_cl _was on an average 1.2 clo (0.186 m²K.W^-1^) and 1.3 clo (0.202 m²K.W^-1^) in mild (-5 to -3 °C) and cold (-12 to -8 °C) conditions, respectively. In addition, the thermal insulation of the clothing was greatly lower in the legs than in the torso.

## Discussion

The thermal sensation "cool" may be experienced unpleasant, but it is not considered to cause harmful cooling or frostbites. If worker has "cold" thermal sensation the risk of harmful cooling should be considered. The questionnaire study revealed that the "cold" thermal sensations increased when ambient temperature was lower than -10 °C. Similarly, workers wore warmer clothing which was sufficient for moderate or heavier physical activity according to required clothing insulation index (IREQ, EN ISO 11079). The field experiment showed that thermal insulation of the clothing was not sufficiently adjusted and sweating moisture condensed into clothing during heavy activity. Heat loss was the highest from legs due to lower thermal insulation than on upper body.

## Conclusion

The open-pit miners' selected their winter clothing based on occupational, environmental and individual factors. The questionnaire based evaluation of basic thermal insulation of the winter clothing provided similar results as measured values during working outdoors in open-pit mines. The results are used for development of the miners' cold protective clothing as well as improvement of the occupational health and safety in the Arctic mining.

## Acknowledgements

This study (http://www.minehealth.eu) was financed by the European Union, Kolarctic ENPI CBC.

